# The Association Between Hepatocellular Carcinoma and Gastrointestinal Adenocarcinoma: Is This a New Syndrome in Patients With Cirrhosis? A Case Series

**DOI:** 10.1002/cnr2.70182

**Published:** 2025-05-09

**Authors:** Fabrizio Bronte, Fabio D'Amato, Maria Rosa Barcellona, Giuseppe Bronte, Giuseppe Malizia, Salvatore Ialuna, Giorgio Fusco, Francesco Verderame, Enrico Bronte, Maria Grazia Bavetta

**Affiliations:** ^1^ Gastroenterology Unit Ospedali Riuniti Villa Sofia – V. Cervello Palermo Italy; ^2^ Interventional Radiology and Neuroradiology Unit Ospedali Riuniti Villa Sofia – V. Cervello Palermo Italy; ^3^ HepatOncology Unit Ospedali Riuniti Villa Sofia – V. Cervello Palermo Italy; ^4^ Department of Translational Medicine University of Ferrara Ferrara Italy; ^5^ Department of Oncology University Hospital of Ferrara Ferrara Italy; ^6^ Nuclear Medicine Unit Ospedali Riuniti Villa Sofia – V. Cervello Palermo Italy; ^7^ Oncology Unit Ospedali Riuniti Villa Sofia – V. Cervello Palermo Italy

**Keywords:** adenocarcinoma, cirrhosis, gastrointestinal, hepatocellular carcinoma

## Abstract

**Aim:**

This case series aimed to explore the occurrence of synchronous hepatocellular carcinoma (HCC) and gastrointestinal adenocarcinoma in cirrhotic patients and to propose a potential common pathogenic mechanism.

**Cases:**

We reviewed the available literature and retrospectively analyzed seven cases of cirrhotic patients with synchronous HCC and gastrointestinal adenocarcinoma (colon or gastric) identified in our center between March 2020 and June 2023. All patients underwent upper gastrointestinal endoscopy, abdominal ultrasound, computed tomography (CT) scan, and histological confirmation through biopsy or surgery.

The mean age of the patients was 77.3 years (range 76–83), with five males and two females. Five patients had liver cirrhosis, and two had chronic hepatitis (one with HCV, one with MASLD). HCC was confirmed in all patients, with elevated alpha‐fetoprotein levels (mean: 737.6 ng/mL). Colon adenocarcinoma was found in five patients, and gastric adenocarcinoma in one patient. Genetic and microsatellite instability analyses were performed in selected cases, revealing high microsatellite instability in one patient. We suggest that the Wnt/APC/β‐catenin pathway might play a key role in the pathogenesis of both HCC and gastrointestinal malignancies.

**Conclusions:**

Synchronous HCC and gastrointestinal adenocarcinoma may be increasingly identified due to prolonged survival in cirrhotic patients. Alterations in the Wnt/APC/β‐catenin pathway could represent a shared pathogenic mechanism. Regular surveillance through ultrasound and endoscopy is essential for early diagnosis in this high‐risk population. Future research is needed to confirm these findings and explore targeted treatments.

**Abbreviations**
APC: adenomatous polyposis coli gene
AXIN1: axis inhibitor 1
AXIN2: axis inhibitor 2
B‐RAF: v‐raf murine sarcoma viral oncogene homolog B1
CA: colon adenocarcinoma
CA19‐9: carbohydrate antigen 19–9
CEA: carcinoembryonic antigen
CT: computed tomography
CTNNB1: catenin beta 1
GSK3B: glycogen synthase kinase‐3 beta
HBV: hepatitis B virus
HCC: hepatocellular carcinoma
HCV: hepatitis C virus
K‐RAS: Kirsten rat sarcoma virus
LEF: lymphoid‐enhancing factor
MASLD: metabolic dysfunction‐associated steatotic liver disease
MetALD: metabolic and alcohol‐associated liver disease
MYC: myelocytomatosis oncogene
N‐RAS: neuroblastoma RAS viral oncogene homolog
RFTA: radiofrequency thermoablation
SARS‐COV‐2: severe acute respiratory syndrome coronavirus 2
TCF: T‐cell factor
U/ml: units/ml
WNT: wingless‐related integration site

## 1 Introduction

Recently, patients with cirrhosis underwent a progressive reduction in hepatocellular carcinoma (HCC) incidence. Etiological therapies, such as those suppressive for the hepatitis B virus (HBV) and eradication therapies for the hepatitis C virus (HCV) contributed to this achievement [1, 2]. This intervention changed the natural history of chronic liver disease, favoring a longer survival for these patients [3]. This phenomenon has highlighted the need for vigilance in monitoring other tumors, including neoplasms in different anatomical regions, such as those affecting the gastrointestinal tract. However, HCC remains the leading cause of death in patients with chronic liver disease, and the risk of liver metastases remains very low [4].

We reviewed the available literature and found only three cases of association of HCC and colon adenocarcinoma (CA) [5, 6, 7]. The first case concerns a patient, 46‐year‐old man, who was diagnosed with HCV‐related liver cirrhosis with a focal liver lesion compatible with HCC and who underwent a liver transplant because he met the Milan criteria. During pre‐transplant screening, the authors performed a colonoscopy, which highlighted a 2 cm lesion of the sigmoid colon compatible with CA with infiltration in 5 out of 18 mesenteric lymph nodes as well as the bowel wall up to the serosa (stage Dukes' C), according to the histologic exam on sigmoidectomy [5]. The second case concerns a patient, 75‐year‐old woman, with HCV liver cirrhosis complicated by bifocal HCC, treated with radiofrequency thermoablation (RFTA). She presented with an HCC recurrence 6 years after the treatment. Given an increase in CEA and CA19‐9, the authors performed a colonoscopy with evidence of a descending colon lesion. After colectomy, the histologic exam was compatible with moderately differentiated tubular CA lacking lymphovascular invasion and stage IIA (T3N0M0) [6]. The third case concerns a 78‐year‐old woman, who has been diagnosed with CA and has undergone hemicolectomy and chemotherapy, and who developed a liver lesion suspicious for metastasis 2 years after treatment. However, the histologic exam on hemihepatectomy was compatible with lymphocyte‐rich HCC characterized by infiltration of lymphocytes among the liver tumor cells (Table 1) [7].

**TABLE 1:** Tumor features in the patients from literature and from our center.

	Case	Markers	HCC (cm)	HCC site	HCC radiology	HCC (Edmondson)	Gastrointestinal (cm)	Gastrointestinal site	Gastrointestinal histology	Stage	RAS/BRAF	Microsatellite instability	Herceptest
Literature review	A (ref. [4])	Not reported (NR)	3.5	S7	Not reported	Not reported	2	Sigmoid	Colon Adenocarcinoma	Not reported	Not reported	Not reported	Not reported
	B (ref. [5])	Alfaphetoprotein: 3 ng/mL CEA: 12.6 ng/mL CA19.9: 65.6 U/mL		Disseminated	Not reported	Not reported	4.5 × 3.5	Caecum	Moderately‐differentiated tubular adenocarcinoma lacking lymphovascular invasion	Not reported	Not reported	Not reported	Not reported
	C (ref. [6])	Alfaphetoprotein: 3 ng/mL CEA: 3.06 ng/mL CA19.9: NR	3.6	S7	Heterogeneous wash‐in Wash‐out portal phase	Lymphocyte‐rich HCC	Not reported	Not reported	Not reported	Not reported	Not reported	Not reported	Not reported
Our cases	1	Alfaphetoprotein: 143 ng/mL CEA: not available (NA) CA19.9: NA	3.6 × 3.2	S6	Poor wash‐in wash‐out	Trabecolar HCC moderately differentiated (II/III)	1.5	Caecum	Colon Adenocarcinoma	Stage IVa (T_4b_ N_x_ M_1a_)	K‐RAS N‐RAS B‐RAF Not mutated	Stable	Not reported
	2	Alfaphetoprotein: 4668 ng/mL CEA: 1.5 ng/mL CA19.9: 5.8 U/mL	7	S8	Poor wash‐in Wash‐out	—	—	Ascending	Colon Adenocarcinoma	Stage II A (pT_3_ N_0_ M_0_)	Not reported	High instability	Not reported
	3	Alfaphetoprotein: 188.2 ng/mL CEA: 6.8 ng/mL CA19.9: 0.2 U/mL	7 × 6 2.5 × 2.3	S8 S5	Wash‐in Wash out	Trabecolar HCC moderately differentiated (I)	—	Ascending	Colon Adenocarcinoma	Stage II A (pT_3_ N_0_ M_0_)	K‐RAS N‐RAS B‐RAF Not mutated	Stable	Not reported
	4	Alfaphetoprotein: 61.3 ng/mL CEA: 3.4 ng/mL CA19.9: 7.3 U/mL	1.5	S6	Wash‐in Wash‐out	—		Right colic flexure	Colon Adenocarcinoma	Stage I (T_1_ N_0_ M_0_)	Not reported	Not reported	Not reported
	5	Alfaphetoprotein: 3.4 ng/mL CEA: 5.6 ng/mL CA19.9: NA	3 × 2.4 4.3 × 4.3	S4 S5	Wash‐in Wash‐out	—		Pyloric antrum	Gastric adenocarcinoma	Stage IV (T_x_ N_1_ M_1_)	Not reported	Not reported	Negative

	6	Alfaphetoprotein: 94.7 ng/mL CEA: 2.3 ng/mL CA19.9: 1.2 U/mL	1.8	S8	Wash‐in Wash‐out	Not reported	7 × 5	Rectum‐sigmoid	Colon Adenocarcinoma	Stage I (T_1_ N_0_ M_0_)	Not reported	Not reported	Not reported
7	Alfaphetoprotein: 4.56 ng/mL CEA: NA CA19.9: NA	2 1.5	S6 S7	Wash‐in Without Wash‐out	—	10	Anus	Colon Adenocarcinoma	Stage IV (cT_3_ N0 M_1_)	Not reported	Not reported	Not reported

According to these EASL 2024 Clinical Practice Guidelines on the management of hepatocellular carcinoma (HCC), patients with established cirrhosis are at a significantly elevated risk of developing HCC. As such, they are recommended to undergo ultrasound‐based surveillance every 6 months. The addition of alpha‐fetoprotein (AFP) measurement may further enhance detection rates. This surveillance interval is supported by evidence demonstrating improved early‐stage tumor identification, which is associated with better clinical outcomes. Importantly, the guidelines emphasize that this approach is applicable to individuals who may benefit from curative‐intent therapies. Conversely, patients with advanced disease and limited life expectancy unrelated to HCC, or those ineligible for curative interventions (e.g., Child‐Pugh class C cirrhosis without access to liver transplantation), are unlikely to derive significant benefit from such surveillance. For patients with advanced fibrosis without overt cirrhosis, routine ultrasound surveillance is not currently recommended due to insufficient data on risk stratification and cost‐effectiveness. While these recommendations provide a broad framework, they are grounded in robust evidence and represent the current standard of care. Ongoing research into risk stratification tools and emerging modalities, such as abbreviated MRI and novel serum biomarkers, may offer future opportunities for more individualized surveillance strategies. However, current data are insufficient to support their integration into routine practice. Prospective studies are anticipated to refine these guidelines further [8]. It is worth noting that while testing for HBsAg and anti‐HCV does not contribute directly to HCC surveillance in patients with cirrhosis, it remains crucial for determining the etiology of chronic liver disease and guiding the initiation of specific treatments. However, there remain some differences between practice guidelines and real‐life clinical practice [9].

In our Unit, 208 patients diagnosed with liver cirrhosis and hepatocellular carcinoma are constantly followed, defined according to current European Association for the Study of the Liver (EASL) guidelines.

The etiology of chronic liver disease is varied and is distributed as follows: 11% of patients present with chronic HBV infection (23 patients), of which 12 have an association with other etiologies (5 with HCV coinfection, 1 with HCV/alcohol, 1 with alcohol, 4 with metabolic disease, 1 with autoimmune disease); 48% of patients have chronic HCV infection (100 patients), of which 18 have an association with other etiologies (5 with HBV coinfection, 11 with alcohol, 1 with metabolic pathology, 1 with hemochromatosis); 12% have alcoholic etiology (25 patients), of which 16 have an association with other etiologies (1 with HBV, 1 with HBV/HCV, 11 with HCV, 3 with metabolic pathology, 1 with hemochromatosis); 21% have metabolic etiology (43 patients), of which 7 are associated with other etiologies (4 with HBV, 1 with HCV, 3 with alcohol); 1.4% have autoimmune etiology (3 patients), of which 1 has HBV coinfection; 1% of patients have hemochromatosis (2 patients) and 5.8% have an undefined etiology (12 patients). Regarding the functional class according to the Child‐Pugh score, 52% of patients (109) fall into class A, 36% (75 patients) into class B, and 12% (24 patients) into class C.

## 2 Case Description

Here, we report a case series of synchronous HCC and gastrointestinal malignancies. We also propose a common etiopathogenetic explanation for the combination of these two neoplasms.

Between March 2020 and June 2023, we identified seven cases of hepatocellular carcinoma synchronous to gastrointestinal, namely colon or gastric, adenocarcinoma. The patients were referred to our center (Ospedali Riuniti Villa Sofia—V. Cervello, Palermo, Italy) for the detection of focal liver lesions. The diagnosis of hepatocellular carcinoma (HCC) was based on the imaging (ultrasound, TC and MR) modalities demonstrating typical features (arterial‐phase hyper‐enhancement and washout in the portal or delayed phases). In cases where imaging findings are inconclusive for the diagnosis of primary liver cancer, liver biopsy was performed to obtain histological confirmation [8]. Only one of these was hospitalized for anemia. The mean age of patients was 77.3 years old (range 76–83), 5 males and 2 females. Two patients had chronic hepatitis (one HCV infection and one MASLD), five were cirrhotic, and the majority were well compensated, three Child‐Pugh A5 and two Child‐Pugh B8 (one patients with MetALD, one of these in combination with HBV and one HBV infection). The two patients classified as chronic hepatitis presented a liver stiffness greater than 12.5 kPa: patient 2 with a value of 23.6 kPa and patient 3 with 18.3 kPa. Both, however, show no ultrasonographic or endoscopic signs of portal hypertension. This picture is indicative of advanced fibrosis (stage F3‐F4), associated with an increased risk of developing HCC [10]. Of note, patient 3 also has metabolic disease, an additional risk factor for HCC. For metabolic etiology, we used the latest nomenclature on fatty liver disease [11]. All patients had cardio‐metabolic comorbidities (four patients had hypertension, two type 2 diabetes mellitus, one hypercholesterolemia and one atrial fibrillation and congestive heart failure, one sinus bradycardia and one with chronic cerebrovascular disease and carotid atherosclerosis) (Table S1).

At the time of the first visit, the patients underwent upper gastrointestinal endoscopy for portal hypertension staging (all patients had no esophageal or gastric varices, only two had portal hypertension gastropathy) and abdominal ultrasound for surveillance, and at least one focal lesion compatible with a primary liver tumor was found in all of them. For this reason, they underwent staging via a computed tomography (CT) scan. CT scans confirmed liver tumor with contrast‐enhancement features suggesting primary liver tumors in three patients, whereas in three patients metastatic lesions were suspected. In these three cases, the patients underwent ultrasound‐guided biopsy of the liver lesion. In two cases, the tumor was histologically a moderately differentiated trabecular HCC (one grade II/III and one grade I according to Edmondson‐Steiner grading system); in one of these, the grade was not reported.

**FIGURE 1:**
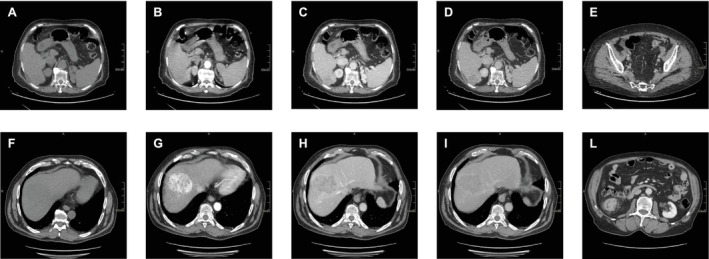
CT scans of patients with diagnosis of moderately differentiated trabecular HCC. Scans of case 1 patient showing HCC in basal (A), arterial (B), venous (C) and late (D) phases, and colon carcinoma (E), and scans of case 3 patient showing HCC in basal (F), arterial (G), venous (H) and late (I) phases, and colon carcinoma (L).

In two cases, the tumor was histologically identified as a moderately differentiated trabecular HCC, with one lesion classified as Grade II/III and another as Grade I according to the Edmondson‐Steiner grading system; however, the grade was not reported for one of these tumors. To provide a comprehensive visualization, we report CT scans (basal, arterial, venous, and delayed phases) (Figure 1).

We reported basal alpha‐fetoprotein (Table 1), and the mean level was 737.6 ng/mL (range: 3.4–4668 ng/mL). Alphafetoprotein is not a diagnostic marker but is a complementary analysis for the diagnosis of hepatocellular carcinoma. It has limited value in the early diagnosis of HCC; the sensitivity of AFP is low (54%) with diagnostic levels in only 18% of patients, but it can be useful as a prognostic indicator in some subgroups of patients. Elevated AFP levels have been correlated with larger tumor size, advanced stages (Okuda and TNM), portal thrombosis, and extrahepatic metastasis. It can be used to evaluate the response to locoregional and systemic treatments. Integration with other diagnostic methods, such as imaging, remains essential for optimal management of HCC [12]. Five of these patients underwent colonoscopy for CT evidence of colonic thickening, whereas one of them underwent gastroscopy for severe anemia, and one underwent colonoscopy for rectorrhage. In all patients with colonic thickening, we found a lesion (two in ascending colon, one in the caecum and one in the right colic flexure, one rectus‐sigmoid) that was histologically compatible with intestinal adenocarcinoma. In the only patient who underwent upper endoscopy, we found an antral lesion compatible with gastric adenocarcinoma. In the patients with rectorrhage, we found an anal lesion histologically compatible with intestinal carcinoma. The mean levels of CEA and CA19‐9 were 5.3 ng/mL (range: 1.5–6.8) and 2.9 U/mL (range: 0.2–7.3), respectively. Genetic analyses for K‐RAS, N‐RAS, and B‐RAF, as per clinical practice, were conducted for two of the six patients, and the results were negative. Microsatellite instability analysis was conducted on three of the six patients, and it showed high instability in one of the three patients. Only in the patient with gastric cancer was the Hercept test conducted, and this resulted in a negative outcome (Table 1). Patients underwent the following treatments for adenocarcinoma and hepatocellular carcinoma: case 1 was treated with systemic fluorouracil for adenocarcinoma and trans‐arterial radioembolization (TARE) for HCC; case 2 had been initially treated for HCC with two TARE sessions and subsequently with Pembrolizumab for adenocarcinoma; case 3 had undergone right hemicolectomy followed by TARE, then followed by sorafenib, and for evidence of neoplastic disease progression, started regorafenib; case 4 underwent an extended right hemicolectomy and subsequently TARE for HCC; case 5 was treated exclusively with capecitabine; case 6 was surgically treated with left hemicolectomy for adenocarcinoma and subsequently with radio‐frequency thermal ablation (RFTA) for HCC; case 7 was undergoing radiotherapy but could not be treated for HCC due to liver failure (Table 2).

**TABLE 2:** Treatment for intestinal adenocarcinoma (IA) and hepatocellular carcinoma (HCC).

Case	Adenocarcinoma treatment	Hepatocellular carcinoma treatment
1	5‐FU	TARE
2	Pembrolizumab	TARE
3	Right hemicolectomy	TARE + Sorafenib + Regorafenib
4	Extendend Right hemicolectomy	TARE
5	Capecitabin	—
6	Left hemicolectomy	RFTA
7	Radiotherapy	—

## 3 Discussion

To the best of our knowledge, and on the basis of our current literature review, just three cases with both HCC and CA have been described till now. The first one had HCC and was subsequently diagnosed with CA during pre‐transplant colonoscopy. The second one had a recurrence of HCC with an increase of CEA and CA19.9, which induced the suspicion of CA and subsequent diagnosis of this malignancy. The latter had a CA diagnosis and a suspected liver metastasis, which then resulted in HCC [5, 6, 7].

The majority of our patients (5 out of 7) in this case series had evidence of colonic thickening in the CT scans, which required a colonoscopy. In the other two, hemorrhage (one for anemia, one for rectorrhage) induced the suspicion, which required endoscopy. This means that the path leading to the double malignant diagnosis can be unpredictable. For this reason, a deeper knowledge of this phenomenon is needed.

Then, we searched for literature to find a potential mechanistic explanation. After a revision of literature, we think that a possible common mechanism for the occurrence of these two kinds of neoplasms can be identified in an alteration of the WNT/APC/β‐catenin pathway. Under normal conditions of the Wnt pathway, β‐catenin interacts with a protein complex formed by APC, AXIN1, AXIN2, phosphate protein 2, and GSK3B. This complex phosphorylates the terminal serine and threonine residues of β‐catenin (encodes by gene CTNNB1) and causes their ubiquitination with subsequent degradation by the proteasome. In the presence of the Wnt ligand that interacts with the membrane receptor Frizzled, however, the protein complex is inhibited, and β‐catenin is not degraded. Then, β‐catenin accumulates in the cytoplasm and enters the nucleus, where it interacts with many transcription factors, including those of the TCF (T‐cell factor)/LEF (lymphoid enhancing factor) family, leading to the activation of the responsive genes of the Wnt pathway. In general, the activation of β‐catenin triggers mechanisms of growth and resistance to apoptosis by activating c‐MYC genes and cyclin D1 [13]. This pathway is mainly impaired in various malignancies, including colorectal cancer, gastric adenocarcinoma, and HCC [14]. As early as 1998, de La Coste et al. reported that APC mutation, and consequent dysregulation of the Wnt/APC/beta‐catenin pathway, was present in 26% of human HCC and 50% of mouse HCC, as well as in colorectal tumors. Specifically, the intracellular concentration of beta‐catenin was elevated. The authors explained that such accumulation of wild‐type β‐catenin can be explained by a mutation of the APC gene, such as in colon cancers, and this was not frequent in HCC because no predisposition to HCC was found in patients with polyposis. Familial adenomatous disease carrying a germline mutation of the APC gene shows that there appears to be no germline genetic alteration at the APC locus in HCC [15]. However, the role of Wnt signaling in liver regeneration, precancerous lesions, and liver cancer has been recognized, as recently reviewed [16, 17]. CTNNB1 and AXIN1 mutations can be found in patients with advanced HCC. Direct targeting of Wnt signaling has been taken into account for cancer therapy. But, the role of Wnt signaling in tissue homeostasis and regeneration can induce severe adverse effects from Wnt blockade, so that specific targets in the pathway should be identified. This knowledge from the literature helped us to generate a hypothesis about the association in the pathogenesis of these malignancies, which will be investigated in further studies.

## 4 Conclusions

Cases of concurrent hepatocarcinoma and gastric or intestinal adenocarcinoma are rarely described in the literature. In this case series, we aimed to provide a detailed analysis of the clinical, radiological, and available histological features of both tumor types in patients experiencing both these diagnoses. We tried to propose a potential shared pathophysiological mechanism underlying their development. We suggest that, with the increase of survival in patients with cirrhosis, synchronous HCC and an adenocarcinoma of the gastrointestinal tract can be found. Chronic inflammation induced by virological and metabolic factors (present in all seven reported cases) could be the basis for the initiation of the carcinogenic process. Our finding in this case series lets us hypothesize that the onset of these malignancies could be associated. Based on the literature, we suppose a common etiopathogenetic mechanism involving the Wnt/APC/beta‐catenin pathway, like a new “cancer syndrome” even though our observations are not sufficient to test this hypothesis. Anyway, further studies could take into account this supposition, and potential treatments targeting common pathways could be developed.

## Author Contributions

Fabrizio Bronte: conceptualization; methodology; data curation; writing – original draft; writing – reviewing and editing. Fabio D'Amato: data curation; validation; writing – reviewing and editing. Maria Rosa Barcellona: data curation; methodology; writing – reviewing and editing. Giuseppe Bronte: validation; methodology; writing – original draft; writing – reviewing and editing. Giuseppe Malizia: conceptualization; data curation; validation; writing – reviewing and editing. Salvatore Ialuna: data curation; methodology; writing – reviewing and editing. Giorgio Fusco: data curation; methodology; writing – reviewing and editing. Francesco Verderame: validation; writing – reviewing and editing. Enrico Bronte: data curation; methodology; writing – reviewing and editing. Maria Grazia Bavetta: conceptualization; data curation; validation; writing – original draft; writing – reviewing and editing. All the authors read and approved the article.

## Ethics Statement

Informed consent was obtained from the patients involved in this article.

## Conflicts of Interest

The authors declare no conflicts of interest.

## Supporting information


**Table S1.** Clinical features of the patients from literature and from our center.

## Data Availability

The data that support the findings of this study are available on request from the corresponding author. The data are not publicly available due to privacy or ethical restrictions.
