# Supplementary material for: The Association Between Hepatocellular Carcinoma and Gastrointestinal Adenocarcinoma: Is This a New Syndrome in Patients With Cirrhosis? A Case Series
Source: Cancer Rep (Hoboken). 2025 May 9;8(5):e70182. doi: 10.1002/cnr2.70182 (PMC12062874; doi:10.1002/cnr2.70182)
Supplement: Supplementary file 1 — Table S1. Clinical features of the patients from literature and from our center. [file CNR2-8-e70182-s001.docx]

**Supplementary Table 1:**

**Clinical features of the patients from literature and from our center.**

|  | **Case** | **Age** | **Sex** | **Etiology** | **Liver stage (Child-Pugh)** | **Comorbidity** | **Portal hypertension** | **Portal Thrombosis** |
| --- | --- | --- | --- | --- | --- | --- | --- | --- |
| **Literature Review** | A (ref. 4) | 46 | M | HCV | Cirrhosis B/C | Not reported | Not reported | Not reported |
|  | B (ref. 5) | 75 | F | HCV | Cirrhosis | Not reported | Not reported | Not reported |
|  | C (ref. 6) | 78 | F | Criptogenetic | No liver disease | Not reported | Not reported | Not reported |
| **Our Cases** | 1 | 76 | F | HBV/MASLD | Cirrhosis CP B 8 | Hypertension  Type 2 diabetes mellitus | Portal hypertension gastropathy | Partial portal thrombosis |
|  | 2 | 80 | F | HCV | Chronic Hepatitis | Hypertension  Hypercolesterolemia | No portal hypertension | No portal thrombosis |
|  | 3 | 78 | M | MASLD | Chronic Hepatitis | Hypertension | Portal hypertension gastropathy | No portal thrombosis |
|  | 4 | 76 | M | HBV | Cirrhosis CP A 5 | Hypertension  Haemorragic stroke  Heart failure | No portal hypertension | No portal thrombosis |
|  | 5 | 83 | M | MASLD | Cirrhosis CP A 5 | Atrial fibrillation  Congestive heart failure | No portal hypertension | No portal thrombosis |
|  | 6 | 77 | M | MASLD | Cirrhosis CP A 5 | Sinus bradycardia | No portal hypertension | No portal thrombosis |
|  | 7 | 71 | M | MetALD | Cirrhosis CP B 8 | Type 2 diabetes mellitus  Chronic cerebrovascular disease  Carotid Atherosclerosis | No portal hypertension | Neoplastic portal thrombosis |
